# A Unique Approach to Generate Self-Aligned SiO_2_/Ge/SiO_2_/SiGe Gate-Stacking Heterostructures in a Single Fabrication Step

**DOI:** 10.1186/s11671-015-0927-y

**Published:** 2015-05-19

**Authors:** Wei-Ting Lai, Kuo-Ching Yang, Ting-Chia Hsu, Po-Hsiang Liao, Thomas George, Pei-Wen Li

**Affiliations:** Department of Electronics Engineering, National ChiaoTung University, HsinChu, 300 Taiwan; Department of Electrical Engineering, National Central University, ChungLi, 320 Taiwan

**Keywords:** Gate-stacking heterostructure, SiGe channel, Self-aligned, Ge quantum dot

## Abstract

We report a first-of-its-kind, unique approach for generating a self-aligned, gate-stacking heterostructure of Ge quantum dot (QD)/SiO_2_/SiGe shell on Si in a single fabrication step. The 4-nm-thick SiO_2_ layer between the Ge QD and SiGe shell fabricated during the single-step process is the result of an exquisitely controlled dynamic balance between the fluxes of oxygen and silicon interstitials. The high-quality interface properties of our “designer” heterostructure are evidenced by the low interface trap density of as low as 2–4 × 10^11^ cm^−2^ eV^−1^ and superior transfer characteristics measured for Ge-based metal-oxide-semiconductor field-effect transistors (MOSFETs). Thanks to the very thin interfacial SiO_2_ layer, carrier storage within the Ge QDs with good memory endurance was established under relatively low-voltage programming/erasing conditions. We hope that our unique self-aligned, gate-stacking heterostructure provides an effective approach for the production of next-generation, high-performance Ge gate/SiO_2_/SiGe channel MOSFETs.

## Background

Rapid expansion of functional electronic applications for mobile devices and for embedded integrated circuit systems has driven a strong and immediate demand for high-speed, low-power transistors and nonvolatile memories. There is, of course, the relentless reduction of feature sizes for achieving desired device performance. In addition, alternative channel materials with higher carrier mobilities are being sought after as a possible solution for realizing next-generation complementary metal-oxide-semiconductor (CMOS) devices [1–9]. Among the possible choices for high-mobility channel materials, Ge has emerged as a leading contender to replace its well-established counterpart, Si, for post-CMOS transistors. This is thanks to Ge’s superior carrier transport efficiency and enhanced charge confinement capabilities [8–16]. Despite these advantages, the production of Ge MOS field-effect transistors (MOSFETs) has been hampered, in particular, in situations where high-temperature thermal oxidation processes are involved. The key for implementing Ge MOS structures lies in the growth of high-quality Ge on Si heterostructures. Sufficiently low defect densities [8, 10] coupled with the formation of gate dielectric layers over the Ge with satisfactory interfacial and electrical properties are required [13–16]. Both of the above requirements are extremely challenging. One is because of the large lattice mismatch of 4.2 % that exists between Ge and Si, and the second is the lack of a robust native oxide for Ge. Unlike the relatively stable SiO_2_, Ge oxide (GeO_*x*_) is water soluble and thermally unstable. Thus, high densities of interface states (*D*_*it*_) and rough interfaces between the oxide and the Ge are produced, both of which are detrimental for the realization of high-speed Ge MOSFETs.

Recently, we have demonstrated a unique CMOS compatible, self-assembled approach to deliberately grow spherical Ge quantum dots (QDs) of desired sizes. We also place these QDs at desired spatial locations with controlled depths of penetration into Si. This was achieved using the exquisite control available through lithographic nanopatterning and selective oxidation of the nanopatterned SiGe layers [17–20]. Remarkably, such self-aligned heterostructures of SiO_2_/Ge QD/SiO_2_/SiGe shell over the Si substrate can be created in a single fabrication step. In this work, we constructed Ge QD MOS capacitors and Ge floating-dot MOSFETs based on this “designer heterostructure” as well as assessed the interfacial properties of Ge QD/SiO_2_/SiGe channel from both structural and electrical perspectives. Our devices exhibit reasonably low interface trap densities, good switching behavior, and excellent charge storage characteristics.

## Methods

**Fig. 1:**
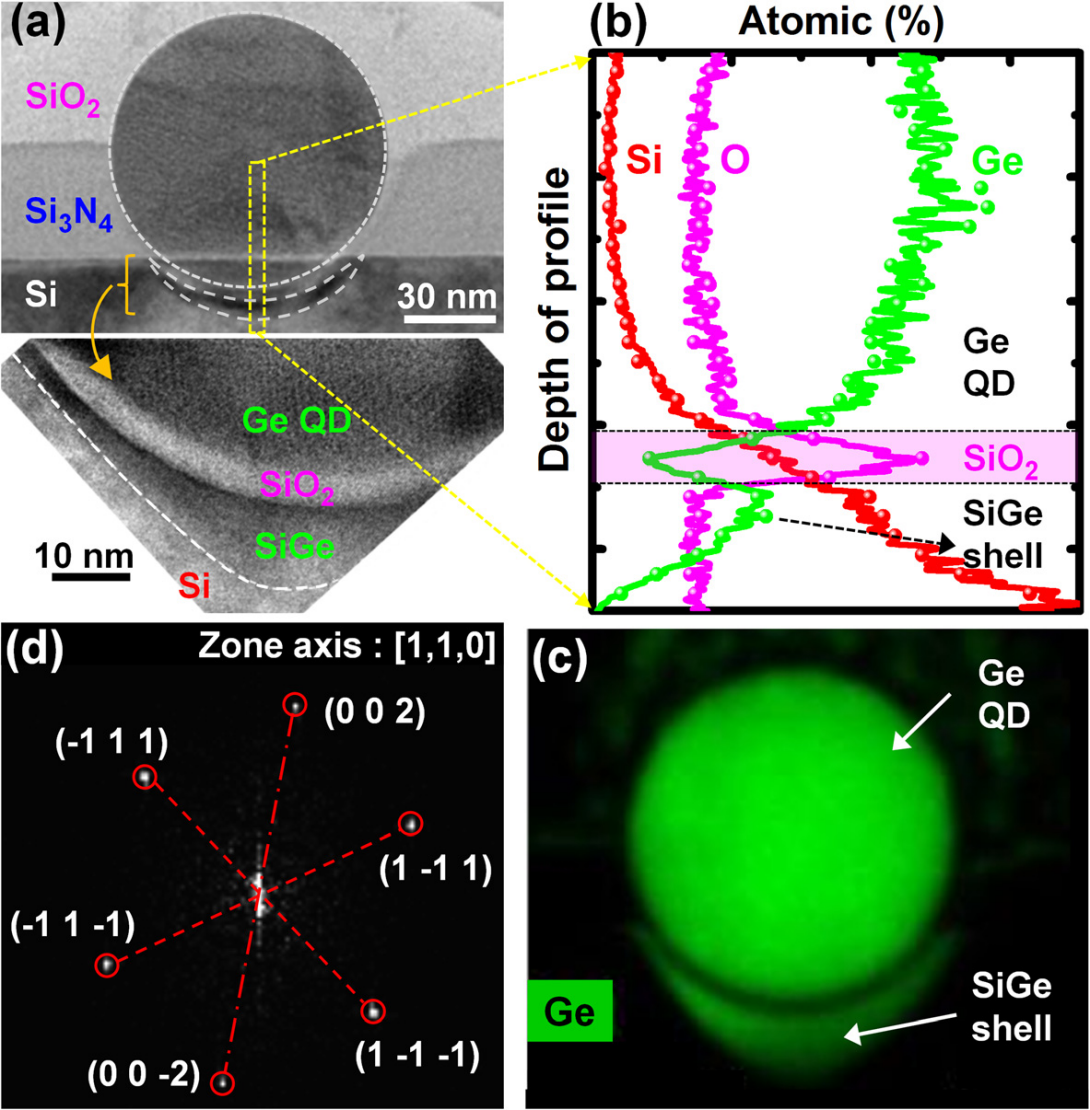
**a** Cross-sectional transmission electron microscopy (CTEM) images as well as EDX elemental **b** line-scan spectra and **c** X-ray mapping micrographs of a SiO_2_/Ge QD/SiO_2_/SiGe shell heterostructure over the Si substrate. **d** Selected area diffraction patterns were generated by applying a fast Fourier transform to the local high-resolution CTEM images of the SiGe shell. The interplanar spacing is approximately 3.16 nm, corresponding to Si_0.28_Ge_0.72_ {111} planes

The fabrication starts with tri-layer, sequential low-pressure chemical vapor deposition of 25-nm-thick Si_3_N_4_, 70-nm-thick poly-Si_0.85_Ge_0.15_, and finally, a capping layer of a 5-nm-thick SiO_2_ over a Si substrate. The topmost, capping SiO_2_ and the poly-Si_0.85_Ge_0.15_ layers are lithographically defined to create 240-nm diameter, nanocylindrical pillars. The SiGe pillar density is approximately 1 × 10^9^ cm^−2^ over the buffer Si_3_N_4_ layers. Next, the nanopatterned structure is subjected to thermal oxidation at 900 °C within an H_2_O ambient producing 90-nm diameter, spherical Ge QDs that migrate into the buffer Si_3_N_4_ layer [17, 18], as shown in Fig. 1a. Next, an Al top gate (with an area of 75 μm × 75 μm) and substrate electrodes are fabricated over the Ge QD array and the backside of the Si substrate, respectively. A final, forming gas anneal at 400 °C completes the fabrication of the Al-SiO_2_/Ge QD/SiO_2_-SiGe MOS capacitors. Additional processes for defining the Al gate and As-doped source/drain (S/D) electrodes are conducted for the fabrication of floating gate MOSFETs. The channel length (*L*_*g*_) and width (*W*) are 3 and 50 μm, respectively. High-resolution, cross-sectional transmission electron microscopy (CTEM) and energy dispersive X-ray (EDX) spectroscopy were used to examine the crystallinity, interfacial morphology, and chemical purity of the Ge QD/SiO_2_/Si heterostructures. Electrical and interfacial properties of SiO_2_/Ge QD/SiO_2_/SiGe MOS devices were measured using frequency-dependent capacitance-voltage(*C*-*V*) and current–voltage (*I*-*V*) characterization over a range of temperatures. Pulse *I*-*V* measurements were also conducted to characterize the floating gate memory devices.

## Results and Discussion

A core building block for floating gate MOS devices is a heterostructure stack consisting of metal-control oxide-floating gate-tunneling oxide-semiconductor, which is usually achieved via multiple-layer, sequential deposition and patterning. Instead, we have succeeded in generating complex heterostructure stacks of 70-nm-thick SiO_2_/90-nm Ge QD/4-nm-thick SiO_2_ over a 20-nm-thick Si_0.28_Ge_0.72_ channel in a single, self-organized nanofabrication step. During the high-temperature oxidation of poly-SiGe nanopillars, the Si content in the nanopillar is preferentially oxidized, squeezing the remaining Ge radially inwards to the centers of the oxidized pillars. Further thermal oxidation resulted in the consolidation of the Ge nanocrystallites in each pillar via Ostwald ripening into single spherical Ge QDs. These QDs also simultaneously migrate through the underlying buffer Si_3_N_4_ layers and achieve contact with the Si substrate, as shown in Fig. 1a. CTEM-EDX line scanning (Fig. 1b) and mapping (Fig. 1c) examinations confirm the high chemical purity of each Ge QD. Intriguingly, a “cup”-shaped morphology is observed for the Ge QD/Si substrate interface (Fig. 1a). The cup is formed by an approximately 4-nm-thick amorphous interfacial oxide layer, conformal with the QD. Below the interfacial oxide layer, an approximately 20-nm-thick Si_1-*x*_Ge_*x*_ shell is generated within the Si substrate. A substantial amount (*x* ≈ 0.72) of Ge as estimated from the Raman signal at 414 cm^−1^ for the SiGe shells formed from the 90-nm Ge QDs [21, 22]. Clear lattice fringes are observed in the high-resolution CTEM micrographs (Fig. 1a). Corresponding selected area diffraction patterns (Fig. 1d), obtained by applying a fast Fourier transform locally to the heterostructure images, confirm the good crystallinity of our self-assembled SiGe shells. The {111} interplanar spacing of ~3.16 nm, suggests that the Si_0.28_Ge_0.72_ shell is under a compressive strain of 2.36 %, thus having great promise for possible high-mobility channel p-MOS applications.

As regards the 4-nm interfacial oxide layer separating the Ge QD and the Si substrate, detailed CTEM-EDX examinations reveal that this amorphous interfacial layer is pure SiO_2_ instead of GeO_*x*_. A sharp dip in the Ge X-ray signal coupled with a simultaneous increase in the Si X-ray intensity is observed from the EDX line scans in Fig. 1b. We have previously proposed [18, 20] that this thin, interfacial SiO_2_ layer between the Ge QD and the SiGe shell is formed by the thermal oxidation of Si interstitials. These interstitials are released from the dissociation of the Si substrate. The SiO_2_ thickness is consequently determined by a dynamic equilibrium that exists between the concentration of Si interstitials and the external oxygen flux. From a chemical thermodynamics perspective, oxygen clearly prefers to react with the Si interstitials rather than with the Ge because of the large difference in enthalpies of formation for SiO_2_ (−910.9 kJ/mole) and for GeO_2_ (−477 kJ/mole) [23]. The clear and abrupt amorphous interfacial SiO_2_ layer between the QD and the SiGe shell suggests an absence of structural defects near either interface, i.e., the Ge QD/SiO_2_ interface as well as the SiO_2_ layer/SiGe shell interface. Therefore, this thermally grown interfacial SiO_2_ layer over the SiGe shell is believed to possess the advantages of robustness, thermal stability, and superior electrical properties. Essentially similar to SiO_2_ thermally grown over Si substrates, our Ge QD/SiO_2_/SiGe heterostructure is amenable for the production of high-performance SiO_2_/SiGe MOS devices.

**Fig. 2:**
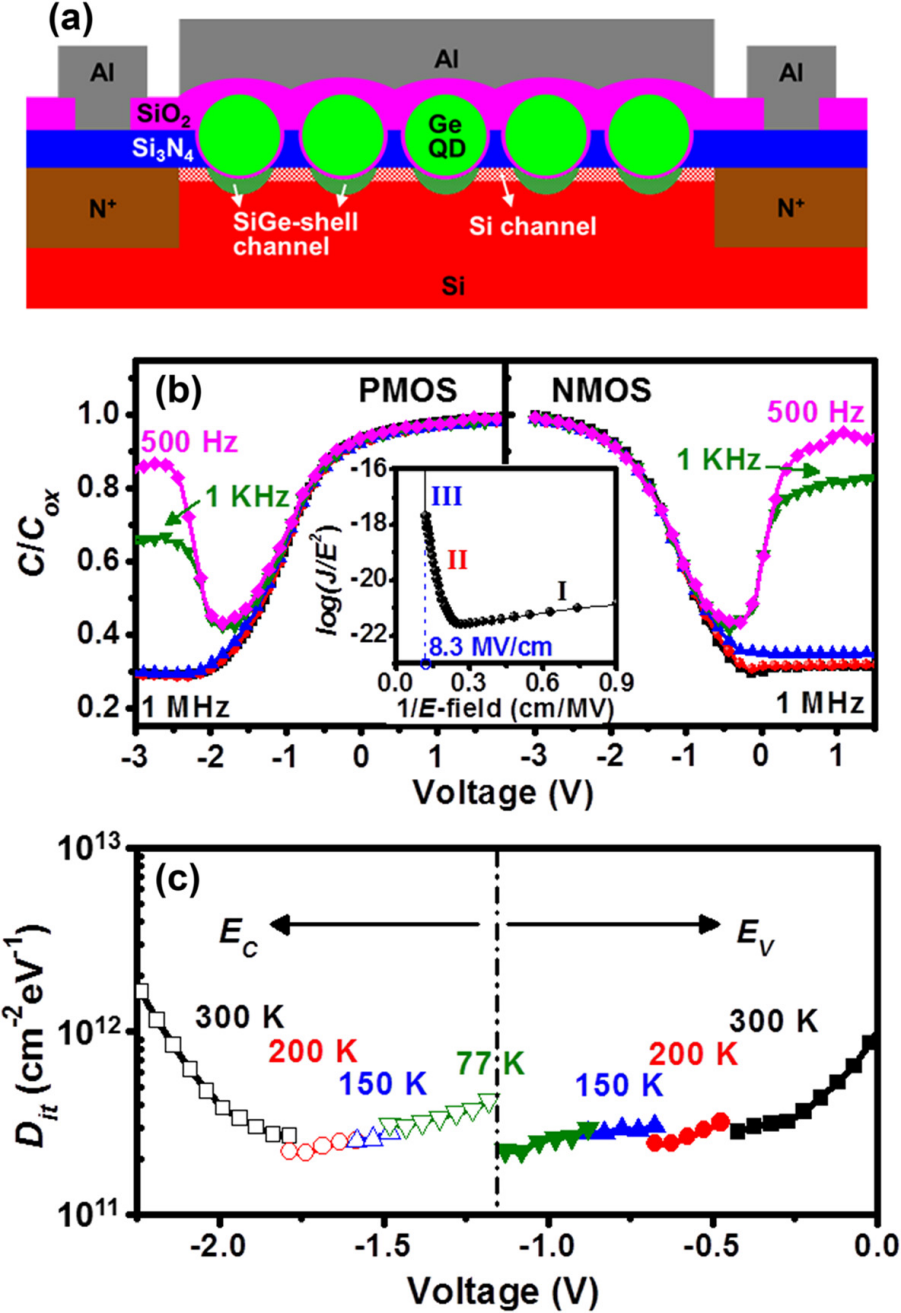
**a** Cross-sectional schematic of the heterostructured SiO_2_/Ge QD/SiO_2_/SiGe n-MOSFET. The gate-stacking region also represents the structural core of the heterostructured MOS capacitor. **b** Frequency-dependent *C*-*V* characteristics of the heterostructured MOS capacitors, where *C*/*C*_*ox*_ represents the normalized capacitance. The inset in **b** shows the corresponding *I*-*V* behavior. **c** *D*_*it*_ values extracted from high-/low-frequency *C*-*V* curves in the temperature range of 300–77 K

Fig. 2a shows the cross-sectional schematic of heterostructured SiO_2_/Ge QD/SiO_2_/SiGe n-MOSFET and MOS capacitor. Notably, the channel of studied heterostructured MOS devices consists of SiGe shell channels below the Ge QDs in series with Si channels with no Ge QDs overhead. The gate dielectric layers of SiO_2_ over the SiGe channel and SiO_2_/Si_3_N_4_ over the Si channel have an equivalent gate oxide thickness (EOT) of approximately 15 and 41 nm, respectively, leading to the SiGe shell being the prime channel that is turned on prior to the parasitic Si channel due to a smaller threshold voltage. Meanwhile, it is a known fact that the interfacial property of Si_3_N_4_/Si channel is not as good as that of SiO_2_/Si. Hence, we believe that our heterostructured SiO_2_/Ge QD/SiO_2_/SiGe MOS devices provide a reasonable platform for the assessment of the interfacial properties of Ge QD/SiO_2_/SiGe channel though extracted interfacial trap density from high-/low-frequency *C*-*V* characteristics of MOS capacitors and from the subthreshold slope (*S.S.*) of transfer (*I*_*d*_-*V*_*g*_) curves for MOSFETs.

*I*-*V* characteristics were measured for the Al-SiO_2_/Ge QD/SiO_2_-SiGe MOS capacitor we fabricated. The typical features of direct tunneling, Fowler-Nordheim tunneling, and hard breakdown, as indicated by regions I, II, and III, respectively, are shown in the inset of Fig. 2b. We also measured a low leakage current density of about 10^−9^ A/cm^2^ and a high breakdown electric field (*E* field) of 8.3 MV/cm. These results verify the electrical robustness of the SiO_2_/Ge QD/SiO_2_/SiGe shell heterostructure. Good gate oxide integrity is further evidenced by its frequency-dependent *C*-*V* characteristics. Fig. 2b clearly shows that the gate bias controls the surface charge and minority carriers very well at 500–1 MHz with almost no frequency-induced dispersion observable for both n- and p-MOS capacitors. Values for mid-gap *D*_*it*_ as low as 2–4 × 10^11^ cm^−2^ eV^−1^ were extracted from the extensive, variable temperature high-/low-frequency *C*-*V* characteristics of more than 20 MOS diodes (Fig. 2c). As mentioned previously, the interfacial property of Si_3_N_4_/Si channel is no better than that of SiO_2_/Si, and thereby, the extracted *D*_*it*_ values of 2–4 × 10^11^ cm^−2^ eV^−1^ may be overestimated for the solely Ge QD/SiO_2_/SiGe channel system. Notably, our results are a sharp contrast to the undesirable frequency- and gate bias-dependent capacitance humps/peaks in the weak inversion region usually observed for conventional GeO_2_/Ge n-MOS capacitors due to the high-interface trap density in the upper half of the bandgap of Ge [11, 15]. Also, our experimental electrical data obviously deviates from the frequency-induced dispersion and stretch out in *C-V* characteristics as well as *E* field-dependent Frenkel-Pool conduction in *I-V* characteristics that are commonly observed for Si_3_N_4_/Si MOS capacitors. Both experimental *C-V* and *I-V* data are further evidence to support the superior, device-quality interface properties and excellent gate oxide integrity of the SiO_2_/Ge QD/SiO_2_/SiGe heterostructure that is applicable for device applications.

**Fig. 3:**
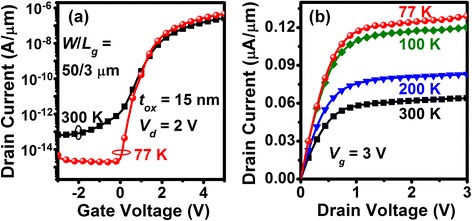
**a** Transfer and **b** output characteristics of the heterostructured SiO_2_/Ge QD/SiO_2_/SiGe n-MOSFETs measured at *T* = 77–300 K. High *I*_on_/*I*_off_ ratio >10^6^ and low off-state leakage of *I*_off_ <10^−13^ A/μm are achieved

*I*_*d*_-*V*_*g*_ of the heterostructured Ge QD/SiO_2_/SiGe channel nMOSFETs show very low off-state leakage of *I*_*off*_ ≅ 10^−13^ A/μm. A superior on-off current ratio of *I*_on_/*I*_off_ >10^6^ and good switching behavior for *S.S.* of 195 mV/dec at *T* = 300 K were also measured (Fig. 3a). This low value of *I*_off_ leakage as well as the high *I*_on_/*I*_off_ ratio is attributable to the high crystalline quality of the SiGe channel and good interface properties for the thin SiO_2_ and SiGe channel. The *I*_*d*_-*V*_*g*_ characteristics were measured at *T* = 77–300 K to estimate *S.S.* from its dependence on temperature. Decreasing the operating temperature from 300 to 77 K results in significant, twofold enhancement of the on-state drive current (Fig. 3b). The *S.S.* improves from 195 to 105 mV/dec as a result of the suppressed phonon scattering. *D*_*it*_ estimated from the *S.S.* value for n-MOSFETs is approximately 4 × 10^11^ cm^−2^ eV^−1^ at *T* = 300 K, which is in good agreement with estimates derived from the high-/low-frequency *C*-*V* characteristics. As mentioned previously, the conducting channel connecting source and drain indeed consists of the prime SiGe shell in series with the second Si inversion layer that would be sequentially turned on due to the difference in the EOT of gate dielectric layers. It would require a greater gate voltage swing (i.e., larger *S.S.*) to completely turn on the entire composited channel than the solely SiGe shell. We envisage that significant improvements in *S.S.* and switching performance are achievable through reducing the parasitic Si channel via shrinking the channel length and increasing the pillar (QD) density within the channel.

Good transfer characteristics such as *S.S.* of ~100–150 mV/dec and low *D*_*it*_ of ~1–5 × 10^11^ cm^−2^ eV^−1^ have been previously reported for the Ge/GeO_2_ or Ge/GeO_2_/high-*k* MOSFETs [9, 11, 14, 15]. However, these characteristics were obtained at the expense of special interfacial treatment prior to and following gate oxidation, such as high-pressure oxidation, two-step oxidation, H_2_O oxidant pre-pulsing, and post-oxidation annealing. Additionally, all the processing temperatures were constrained to be below 500 °C in order to prevent GeO desorption that severely deteriorates the Ge/dielectric interface and the thermal stability of the Ge MOSFETs [9, 14, 15]. In order to meet the temperature constraint requirement for the production of Ge/GeO_2_ or Ge/GeO_2_/high-*k* MOSFETs, a “gate-last” process is employed. This is because the temperature to activate n-type dopants for source/drain is generally, significantly higher than 500 °C. Finally, in spite of achieving good *S.S.* and low *D*_*it*_ values, the low *I*_on_/*I*_off_ (~10^4^) of Ge/GeO_2_/high-*k* MOSFETs is not practicable for low-power device applications because of the high S/D junction leakage due to insufficient activation and rapid diffusion of n-type dopants in Ge [9, 11, 14, 15]. Thus, as compared to the best Ge/GeO_2_ or Ge/GeO_2_/high-*k* MOSFETs, our self-aligned Ge QD/SiO_2_/SiGe gate-stacking heterostructure possesses comparable interfacial properties (*D*_*it*_) and subthreshold swing behavior (*S.S.*). However, the significant advantage of our approach over the conventional state-of-the-art is our fabrication in a single-step, self-organized oxidation process, with the preferred, thermally stable, robust interfacial SiO_2_ layer rather than the unstable GeO_2_. Most importantly, our high *I*_on_/*I*_off_ >10^6^ is simultaneously achieved within our designer Ge QD/SiO_2_/SiGe channel nMOSFETs with S/D junctions that are n^+^/p Si homojunctions just like in conventional Si MOSFETs.

**Fig. 4:**
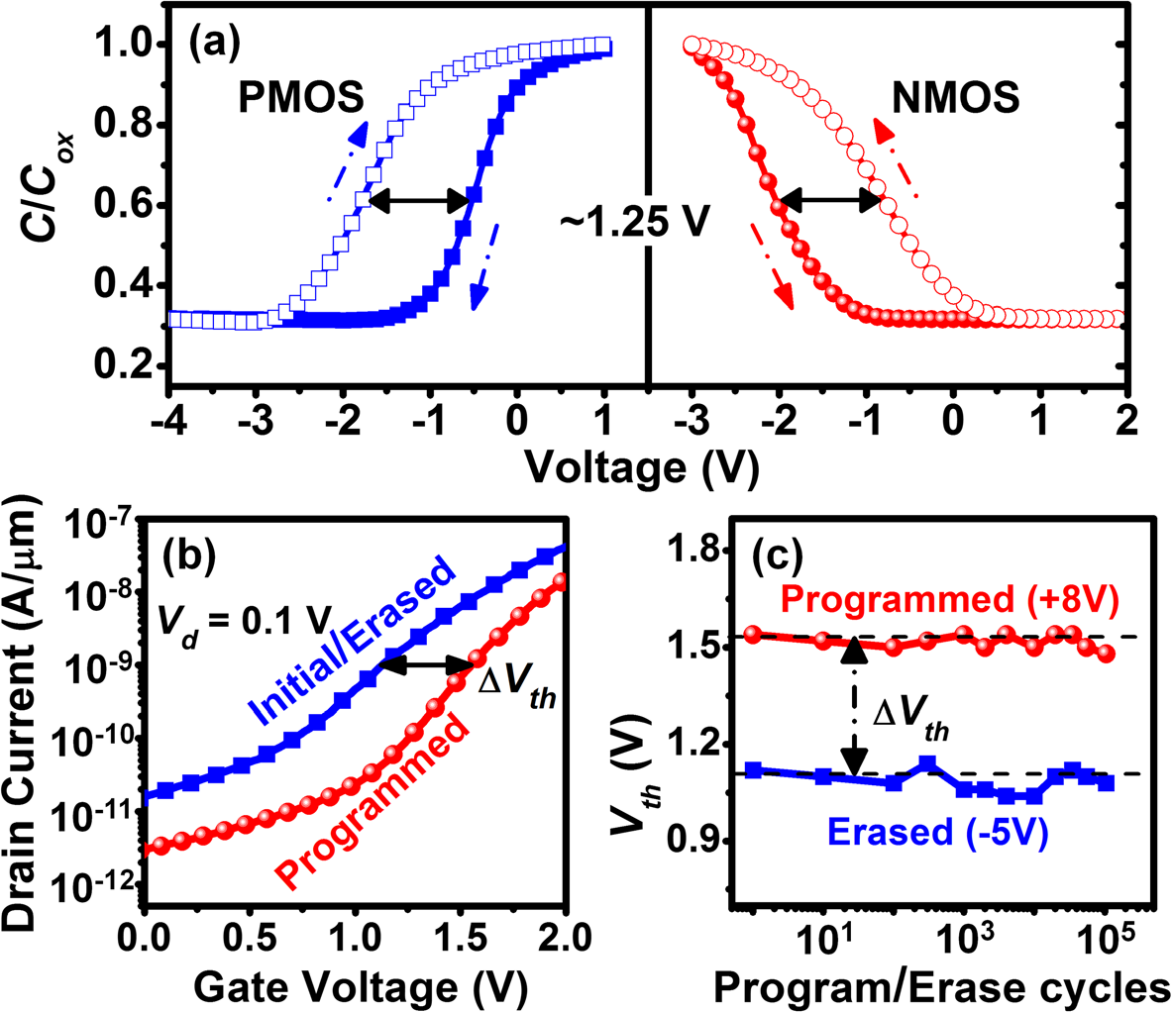
**a** Hysteresis loop characteristics with voltage shift as large as 1.25 V are observed for both n- and p-MOS capacitors when the gate bias is swept from inversion to accumulation conditions. **b** *I*_*d*_-*V*_*g*_ and **c** memory endurance characteristics of Ge QD floating gate memories under a +8 V/60 msec pulse programing and a −5 V/30 msec pulse erasing. The *I*_*d*_-*V*_*g*_ characteristics are swept from 0 to 2 V. Δ*V*_th_ of ~0.42 V is achieved with negligible Δ*V*_th_ degradation following 10^5^ program/erase cycles

As mentioned above, our heterostructured Ge QD/SiO_2_/SiGe channel also provides an effective building block for the realization of Ge nanocrystal-based floating gate memories. Thanks to the considerable barrier height adjacent the Ge QD for confining both electrons (~3.2 eV) and holes (~5 eV), significant charge storage stability is achieved within the Ge QDs. This important property was confirmed by counterclockwise and clockwise *C*-*V* hysteresis loop measurements in n- and p-MOS capacitors for electron and hole storage, respectively (Fig. 4a). The slopes of *C*-*V* curves prior to and following charge injection are nearly unchanged. The nearly indiscernible frequency-induced dispersion and stretch out in *C*-*V* characteristics (Fig. 2a), suggests that the charge is primarily stored within the QDs rather than being captured by interface traps or deep traps within Si_3_N_4_. The Ge QD, floating gate memory effect is further evidenced by a threshold voltage (*V*_th_) shift of Δ*V*_th_ ~ 0.42 V, which was measured by applying gate voltage pulse under low-voltage programming (+8 V/60 msec) and erasing (−5 V/30 msec) conditions, as shown in Fig. 4b. Δ*V*_th_ is defined by the difference in gate voltage for these two conditions at a constant drain current value of 1 nA/μm in channel width. Also, thanks to the very thin, 4-nm-thick tunneling oxide, our Ge QD floating gate memory allows low-voltage programming and erasing at +8 and −5 V, respectively. These voltages are almost one half of the corresponding voltages required for state-of-the-art floating gate memory devices [24–26] and definitely very desirable from a reliability perspective. A good memory endurance of more than 10^5^ cycles with negligible Δ*V*_th_ degradation is measured for our Ge QD devices (Fig. 4c) under the programming/erasing conditions of +8 V/−5 V. The above result is a further demonstration of the good charge storage capability and also confirms the electrical robustness of the thin, interfacial SiO_2_ layer.

## Conclusions

We have demonstrated a unique approach for generating self-aligned SiO_2_/Ge QD/SiO_2_/SiGe shell MOS heterostructures using a single oxidation step of SiGe nanopillars over a buffer Si_3_N_4_ layer on the Si substrate. Further, we have realized Ge QD floating gate memory devices based on this designer heterostructure. Superior interfacial morphologies and high-quality electrical properties of the SiO_2_/Ge QD/SiO_2_/SiGe shell heterostructure have been multiply confirmed by extensive TEM, EDX, *I*-*V*, and *C*-*V* characterization. Thanks to the thin and robust tunneling oxide, charge storage within the Ge QDs produces considerable voltage shifts under relatively low-voltage programing/erasing conditions with good memory endurance characteristics.

Our self-aligned, Ge QD-based, gate-stacking structure is analogous to the prevailing poly-Si/SiO_2_/Si MOS structure and indeed provides a practically achievable core building block for Ge-based MOS devices with size-tunable Ge gates, SiO_2_ gate oxide, and SiGe channels. It is worthwhile to point out the electronic performance advantages of the single-crystalline SiGe shell arising both from having superior interface properties as well as being in a state of compressive stress inherently generated during the fabrication process, which are favorable for the production of high-performance p-MOSFETs. Further device structure design including the pillar (or QD) density and gate area is undergoing for the optimal performance of n- and p-MOSFETs. Thus, we believe that our self-aligned heterostructure is a very promising functional candidate for the realization of next-generation, high-performance SiGe (or Ge) MOSFETs, using the precise control available through our self-organized, single-step fabrication process.
